# Antibiotic prophylaxis in preterm premature rupture of membranes at 24–31 weeks’ gestation: Perinatal and 2‐year outcomes in the EPIPAGE‐2 cohort

**DOI:** 10.1111/1471-0528.17081

**Published:** 2022-01-13

**Authors:** Elsa Lorthe, Mathilde Letouzey, Héloïse Torchin, Laurence Foix L'Helias, Christèle Gras‐Le Guen, Valérie Benhammou, Pascal Boileau, Caroline Charlier, Gilles Kayem, for the EPIPAGE‐2 Obstetric Writing Group, Pierre‐Yves Ancel, Catherine Arnaud, Julie Blanc, Thierry Debillon, Pierre Delorme, Claude D’Ercole, Thomas Desplanches, Caroline Diguisto, Géraldine Gascoin, Catherine Gire, François Goffinet, Bruno Langer, Emeline Maisonneuve, Stéphane Marret, Isabelle Monier, Andrei Morgan, Jean‐Christophe Rozé, Thomas Schmitz, Loïc Sentilhes, Damien Subtil, Barthélémy Tosello, Christophe Vayssière, Norbert Winer, Jennifer Zeitlin, D Astruc, P Kuhn, J Matis, C Ramousset, X Hernandorena, P Chabanier, L Joly‐Pedespan, MJ Costedoat, A Leguen, B Lecomte, D Lemery, F Vendittelli, G Beucher, M Dreyfus, B Guillois, Y Toure, A Burguet, S Couvreur, JB Gouyon, P Sagot, N Colas, J Sizun, A Beuchée, P Pladys, F Rouget, RP Dupuy, D Soupre, F Charlot, S Roudaut, A Favreau, E Saliba, L Reboul, N Bednarek, P Morville, V Verrière, G Thiriez, C Balamou, L Marpeau, C Barbier, X Durrmeyer, M Granier, M Ayoubi, O Baud, B Carbonne, PH Jarreau, D Mitanchez, C Duffaut, L Cornu, R Moras, P Boulot, G Cambonie, H Daudé, A Badessi, N Tsaoussis, A Bédu, F Mons, C Bahans, MH Binet, J Fresson, JM Hascoët, A Milton, O Morel, R Vieux, L Hilpert, C Alberge, M Baron, ML Charkaluk, V Pierrat, P Truffert, S Akowanou, U Simeoni, A Bongain, M Deschamps, B Branger, V Rouger, C Dupont, J Gondry, G Krim, B Baby, M Debeir, O Claris, JC Picaud, S Rubio‐Gurung, C Cans, A Ego, H Patural, A Rannaud, E Janky, A Poulichet, JM Rosenthal, E Coliné, A Favre, N Joly, S Châlons, J Pignol, PL Laurence, PY Robillard, S Samperiz, D Ramful, B Blondel, M Bonet, A Brinis, A Coquelin, M Durox, M Kaminski, K Khemache, B Khoshnood, C Lebeaux, L Marchand‐Martin, J Rousseau, MJ Saurel‐Cubizolles, D Tran

**Affiliations:** ^1^ Université de Paris, Epidemiology and Statistics Research Center/CRESS, INSERM, INRA Paris France; ^2^ Unit of Population Epidemiology, Department of Primary Care Medicine Geneva University Hospitals Geneva Switzerland; ^3^ Department of Neonatal Paediatrics Poissy Saint Germain Hospital, Versailles Saint Quentin en Yvelines University Poissy France; ^4^ Department of Neonatal Paediatrics Cochin Port Royal Hospital, APHP Paris France; ^5^ Department of Neonatal Paediatrics Trousseau Hospital, APHP, Sorbonne University Paris France; ^6^ Department of Paediatrics, Paediatrics Emergency Unit and General Paediatrics Nantes University Hospital, Hôpital Mère‐Enfant, CHU de Nantes Nantes France; ^7^ UFR des Sciences de la Santé Simone Veil Versailles St Quentin en Yvelines University Montigny le Bretonneux France; ^8^ Université de Paris, Hôpital Universitaire Necker‐Enfants Malades, Biology of Infection Unit, Division of Infectious Diseases and Tropical Medicine, Assistance Publique‐Hôpitaux de Paris, Institut Pasteur, French National Reference Centre and WHO Collaborating Centre for Listeria U1117 Inserm U1117 Paris France; ^9^ Department of Gynaecology and Obstetrics Trousseau Hospital, APHP, Sorbonne University Paris France

**Keywords:** amoxicillin, antenatal management, cephalosporins, latency, macrolides, neurodevelopment, obstetric intervention, perinatal outcome, prematurity, prophylactic antibiotics

## Abstract

**Objective:**

To compare different antibiotic prophylaxis administered after preterm premature rupture of membranes to determine whether any were associated with differences in obstetric and/or neonatal outcomes and/or neurodevelopmental outcomes at 2 years of corrected age.

**Design:**

Prospective, nationwide, population‐based EPIPAGE‐2 cohort study of preterm infants.

**Setting:**

France, 2011.

**Sample:**

We included 492 women with a singleton pregnancy and a diagnosis of preterm premature rupture of membranes at 24–31 weeks. Exclusion criteria were contraindication to expectant management or indication for antibiotic therapy other than preterm premature rupture of membranes. Antibiotic prophylaxis was categorised as amoxicillin (*n* = 345), macrolide (*n* = 30), third‐generation cephalosporin (*n* = 45) or any combinations covering *Streptococcus agalactiae* and >90% of *Escherichia coli* (*n* = 72), initiated within 24 hours after preterm premature rupture of membranes.

**Methods:**

Population‐averaged robust Poisson models.

**Main Outcome Measures:**

Survival at discharge without severe neonatal morbidity, 2‐year neurodevelopment.

**Results:**

With amoxicillin, macrolide, third‐generation cephalosporin and combinations, 78.5%, 83.9%, 93.6% and 86.0% of neonates were discharged alive without severe morbidity. The administration of third‐generation cephalosporin or any *E*. *coli*‐targeting combinations was associated with improved survival without severe morbidity (adjusted risk ratio 1.25 [95% confidence interval 1.08–1.45] and 1.10 [95 % confidence interval 1.01–1.20], respectively) compared with amoxicillin. We evidenced no increase in neonatal sepsis related to third‐generation cephalosporin‐resistant pathogen.

**Conclusion:**

In preterm premature rupture of membranes at 24–31 weeks, antibiotic prophylaxis based on third‐generation cephalosporin may be associated with improved survival without severe neonatal morbidity when compared with amoxicillin, with no evidence of increase in neonatal sepsis related to third‐generation cephalosporin‐resistant pathogen.

**Tweetable Abstract:**

Antibiotic prophylaxis after PPROM at 24–31 weeks: 3rd‐generation cephalosporins associated with improved neonatal outcomes.

## Tweetable Abstract

Antibiotic prophylaxis after PPROM at 24–31 weeks: 3rd‐generation cephalosporins associated with improved neonatal outcomes.

## 1 INTRODUCTION

Preterm premature rupture of membranes (PPROM), defined as spontaneous rupture of fetal membranes before 37 weeks’ gestation and before labour, occurs in 3% of pregnancies and is one of the leading causes of preterm birth.^1^, ^2^, ^3^ Intrauterine infection can be both a cause and a consequence of PPROM, exposing the mother, the fetus and subsequently the neonate to increased perinatal morbidity.^1^, ^4^, ^5^ Antenatal management of women with PPROM thus aims to reduce the adverse consequences of intrauterine infection and prematurity.

The positive impact of antibiotic prophylaxis is now well evidenced, with significant reduction of neonatal and maternal morbidity (including neonatal infection, use of surfactant, oxygen therapy, abnormal cerebral ultrasound scan and clinically defined intrauterine infection) compared with placebo in large randomised controlled trials and meta‐analyses,^4^, ^6^ leading to strong recommendations and wide use in clinical practice.^3^, ^7^, ^8^, ^9^, ^10^, ^11^

However, treatment modalities (agent, route of administration and duration) are still being debated. As stated by Mercer et al.,^12^ a number of antibiotics and antimicrobial regimens have been studied to cover ‘the broad spectrum of aerobic and anaerobic bacteria and mycoplasmas that have been implicated as causative agents for intrauterine infection at the time of preterm delivery and PPROM’”. Indeed, a large meta‐analysis that compiled 22 randomised trials evaluating the benefits of antibiotics in PPROM (*n* = 6872), recorded 11 different antibiotic agents (mostly ampicillin/amoxicillin, broad spectrum penicillins, amoxicillin/clavulanate and macrolides), used as a single agent or in combination, intravenously, orally or both, for a duration ranging from 1 to 10 days, or until delivery.^4^ Of them, amoxicillin/clavulanate has now been discarded as it was shown to be associated with an increased risk of necrotising enterocolitis (NEC) in preterm neonates.^13^

The controversial role of mycoplasmas as ‘innocent bystanders’ or pathogens in intrauterine infections,^14^, ^15^ the evolution of bacterial ecology since the 1990s, the current questioning regarding the impact of antibiotic prescriptions on microbiota^16^ and the scarce evaluation of antibiotics such as third‐generation cephalosporins (3GC) point out the urgent need for a re‐appreciation of available antibiotics. We aimed to compare different antibiotic prophylaxis administered after PPROM to determine whether any were associated with differences in obstetric and/or neonatal outcomes and/or neurodevelopmental outcomes at 2 years of age.

## 2 MATERIALS AND METHODS

### 2.1 Study design and participants

This is a secondary analysis of the EPIPAGE‐2 cohort, a prospective national population‐based cohort of preterm infants that included all live born or stillborn infants and all terminations of pregnancy at 22–34 completed weeks’ gestation in all maternity units from 25 regions in France in 2011.^17^ Recruitment took place at birth, after families had received information and agreed to participate. Infants were included at three different periods by gestational age at birth: 8‐month recruitment for births at 22–26 weeks, 6‐month recruitment for 27–31 weeks, and 5‐week recruitment for 32–34 weeks. This recruitment strategy aimed to over‐represent extremely preterm births (22–26 weeks) because of their low incidence and to include only a sample of moderate preterm births (32–34 weeks). Maternal, obstetric and neonatal data were collected from medical records following a standardised protocol as previously reported.^17^ At 2 years of corrected age, children participating in the follow‐up were assessed with a detailed neurological and sensory examination performed by their referring physician, and a standardised questionnaire about development was completed by the parents.^18^ The cohort relies on several sources of funding through grants awarded after external peer review for scientific quality. Recruitment and follow‐ups were approved by the appropriate ethics committees, i.e. the advisory committee on the treatment of personal health data for research purposes (references 10‐626, 12‐109 and 16‐263) and the committee for the protection of people participating in biomedical research (reference n° 2011‐A00159‐32 and 2016‐A0033‐48). All the procedures were in accordance with the Declaration of Helsinki of the World Medical Association.

Patients were not involved in setting the research question or the outcome measures, nor were they involved in developing plans for design of the study. Parents showed overwhelming support for the study through high follow‐up rates. EPIPAGE‐2 maintains contact with parents in the cohort through letters, newsletters and its website. National parents’ associations assisted with results dissemination.

**FIGURE 1:**
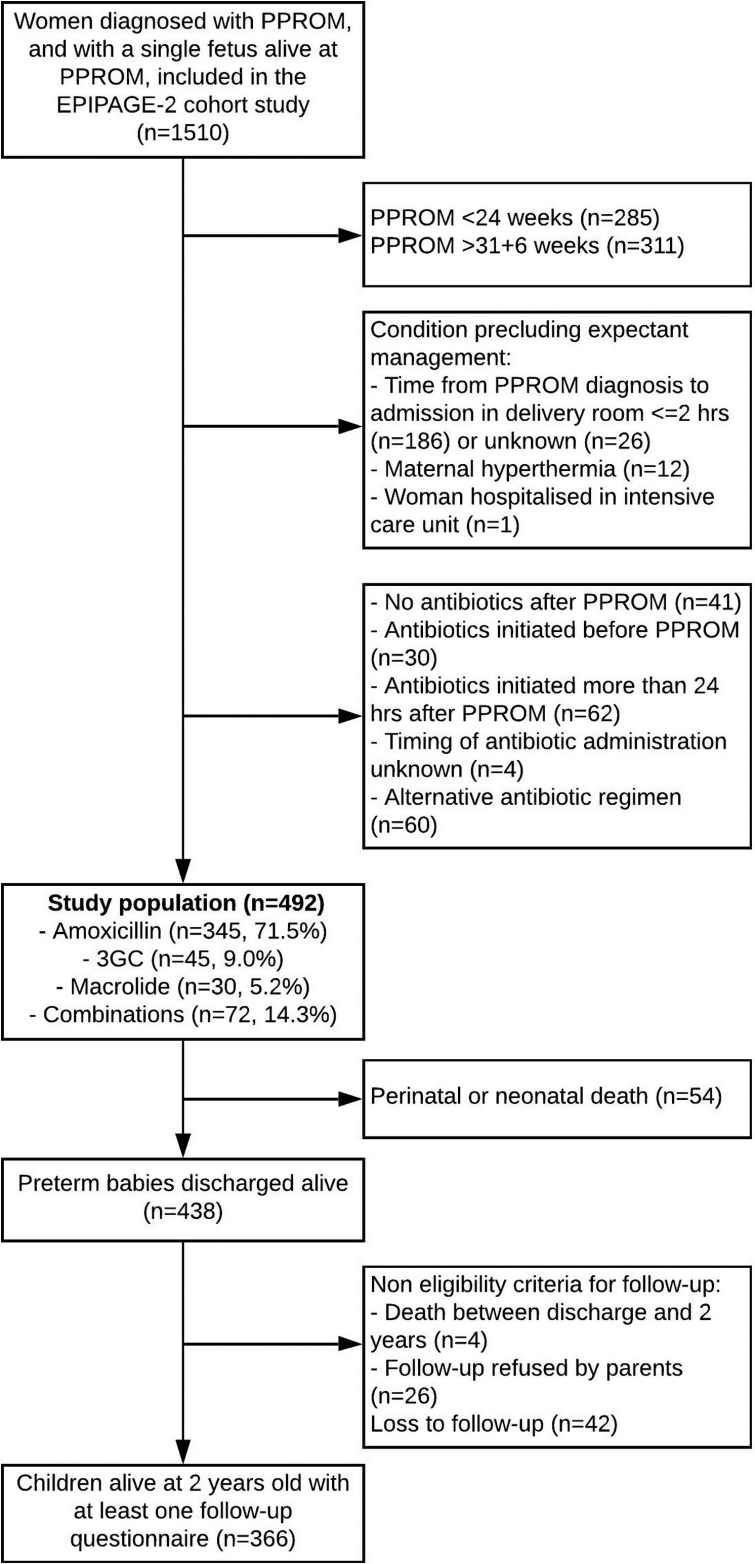
Flow chart. Summarises how sample size of analysis was obtained. PPROM, preterm premature rupture of membranes

Our study population included all women with a singleton pregnancy, PPROM at 24–31 weeks and a non‐malformed fetus who was alive at PPROM diagnosis and born at 24–34 weeks (Figure 1). As recommended, the diagnosis of PPROM was based on maternal history and sterile speculum examination with a diagnostic test if necessary. We excluded women with contraindication to expectant management and/or indication for antibiotic therapy (such as overt intrauterine infection or fever) at diagnosis.

### 2.2 Antibiotic prophylaxis

We excluded women who initiated antibiotics either before PPROM or >24 hours after PPROM and those who received amoxicillin/clavulanate because of the increased risk of NEC (Figure 1). Women who did not receive antibiotics at all received tocolysis and antenatal steroids less often and delivered more quickly, whereas women with an alternative antibiotic regimen overall displayed similar characteristics and outcomes to those analysed (Table S1). For analysis purposes, among the 30 different antibiotic prophylaxis regimens identified, we considered the main families of antibiotic prescribed with sufficient sample size, namely, amoxicillin (*n* = 345), macrolides/clindamycin (*n* = 30: erythromycin *n* = 16, spiramycin *n* = 1, and clindamycin *n* = 13), parenteral 3GC (*n* = 45: ceftriaxone, *n* = 32, cefotaxime, *n* = 13) and any combinations of antibiotics (*n* = 72) covering >90% of *Escherichia coli* (i.e. 3GC and/or an aminoglycoside) in addition to *Streptococcus agalactiae* (i.e. clindamycin, amoxicillin, 3GC or any other β‐lactam) (Table S2). The other antibiotic combinations (*n* = 27) were not investigated.

### 2.3 Outcomes

Perinatal outcomes included survival, i.e. the number of babies discharged alive relative to the number of fetuses alive at PPROM diagnosis, and survival without severe neonatal morbidity.^19^ Severe neonatal morbidity was defined as any of the following: grades III–IV intraventricular haemorrhage, cystic periventricular leucomalacia, stages II–III NEC according to Bell’s staging, stage 3 or greater retinopathy of prematurity according to international classification and/or laser treatment, and severe bronchopulmonary dysplasia at 36 weeks of gestational age.

We further investigated prolongation of gestation and infectious morbidity for both the mother and the neonate. Prolongation of gestation was defined as latency duration (i.e. time from rupture of membranes to delivery) ≥48 hours and ≥7 days. Intrauterine infection was defined as a maternal temperature >37.8°C (100°F) with any two of the following criteria: uterine tenderness, purulent or foul‐smelling amniotic fluid, maternal tachycardia, fetal tachycardia, maternal leucocytosis >15 000 cells/mm^3^.^20^, ^21^ Among newborns, early‐onset sepsis (EOS) was diagnosed by positive bacteriology findings in blood or cerebrospinal fluid beginning in the first 72 hours of life. Late‐onset sepsis (LOS) was defined as a positive blood culture, occurring after 72 hours of life, associated with antibiotic administration for 5 days or more, or death within 5 days following positive blood culture. We were able to report bacterial documentation and resistances in EOS and LOS.

Finally, we studied survival at 2 years’ corrected age (CA) without severe or moderate neuromotor or sensory disabilities (i.e. without gross motor function classification system [GMFCS] level 2–5 cerebral palsy,^22^ blindness and/or deafness), and parental‐reported neurodevelopment at 2 years’ corrected age (assessed with the second version of the 24 months’ Ages and Stages Questionnaire [ASQ]).^18^ We presented both the overall total ASQ score (maximum = 300) and the ASQ score below threshold defined as a score <2 SD from the mean on any of the five domains, for children without cerebral palsy, blindness or deafness, whose parents completed the questionnaire between 22 and 26 months’ corrected age.

In the present study, we report 11 of 12 outcomes included in the core outcome set for neonatal research.^23^ Quality of life was not collected as part of the 2‐year follow‐up evaluation.

### 2.4 Definition of other variables

Socio‐economic position was defined as the highest occupational status of the mother and father, or mother only if a single parent. Gestational age was determined as the best obstetrical estimate combining the last menstrual period and the first trimester ultrasonography assessment. Babies were considered small for gestational age if their birthweight was ≤10th percentile of the normalised *Z*‐score, calculated from French EPOPé intrauterine growth curves adjusted for fetal sex and gestational age.^24^ Maternity units were considered as type 3 when associated with a neonatal intensive care unit (NICU).

### 2.5 Statistical analyses

Maternal and neonatal characteristics and outcomes were described as frequencies and percentages. Percentages were weighted according to the duration of the recruitment periods (in weeks) by gestational age: weights were 1.0 (35/35) for births at 24–26 weeks, 1.34 (35/26) at 27–31 weeks, and 7.0 (35/5) at 32–34 weeks. Weighting allowed us to account for the sampling scheme of the cohort and to ensure representativeness. We then compared characteristics and outcomes by antibiotic prophylaxis regimen using chi‐square or Fisher’s exact tests as appropriate for categorical variables, based on the weighted percentages, and nonparametric equality of medians test for quantitative variables. Survival curves of latency duration (considered as a continuous variable) by antibiotic prophylaxis regimen were plotted using the Kaplan–Meier method and compared with a log rank test.

The antibiotic prophylaxis regimen after PPROM is usually chosen at the unit‐level using a local guideline. Thus, the association between antibiotic prophylaxis regimens and outcomes was evaluated using population‐averaged Poisson regression models, a generalised linear model with a log link and a Poisson distribution, with robust variance estimation, which accounts for the clustering of women within maternity units.^25^, ^26^ Under the population‐averaged model, we obtained the risk of an average woman exposed to an antibiotic prophylaxis regimen presenting the outcome compared with the risk of an average woman exposed to another antibiotic prophylaxis regimen presenting the outcome. Multivariate models were minimally adjusted for gestational age at PPROM and the type of maternity unit, as these two variables were considered confounders. We assumed that other individual characteristics would not be taken into account in choosing a specific antibiotic, except antibiotic allergy but this information was not available. Results are reported as risk ratios (RRs) with 95% confidence intervals (95% CI).

Active antenatal management may differ by gestational age at PPROM, especially with extremely preterm rupture of membranes, and may result in poorer neonatal outcomes. We therefore performed a sensitivity analysis by stratifying on gestational age at PPROM (24–26 vs. 27–31 weeks), except for infectious morbidities, as there were too few of these to adequately run the models.

The proportion of missing data per covariate ranged from 0% to 7% for perinatal data, and reached 43% for the ASQ score because of attrition and further exclusion of ASQ results when parental questionnaires had been filled before 22 months or after 26 months of corrected age. Children who did not participate in the follow‐up at 2 years’ corrected age were born to younger mothers, more often after a complete course of antenatal steroids and in type 3 maternity units (Table S3). We performed multiple imputations with chained equations using baseline and outcomes variables and those potentially predicting nonresponse and/or outcomes (namely, type of maternity unit, maternal age, country of birth, socio‐economic position, parity, gestational age at PPROM, PPROM occurring during hospitalisation for another reason, antibiotic prophylaxis, tocolysis, antenatal steroids, in utero transfer, fetal presentation, fetal sex, small for gestational age, latency duration, mode of delivery, intrauterine infection, vital status at birth, survival at discharge, early‐onset sepsis, late‐onset sepsis, retinopathy of prematurity, severe neonatal morbidity, death between discharge and follow‐up at 2 years of corrected age, survival at 2 years without neurosensory impairment, ASQ score below threshold). Outcomes were estimated within each of the 50 imputed datasets generated with 20 iterations, and results were pooled according to Rubin rules. Statistical significance was set at a two‐tailed value of *P* < 0.05. No correction for multiple comparisons was made, as we aimed to generate hypotheses. Data were analysed using STATA/SE 13.0 (StataCorp LP).

## 3 RESULTS

A total of 492 women with PPROM from 85 maternity units met the inclusion criteria. Of them, 345, 30, 45 and 72 (weighted percentages, 71.5%, 5.2%, 9.0% and 14.3%) received amoxicillin, a macrolide, a 3GC or a combination targeting *E*. *coli* and *S*. *agalactiae*, respectively (Figure 1).

Overall, maternal, obstetric and neonatal characteristics were similar across the four groups (Table 1). Outcomes are described in Table 2 and their association with antibiotic prophylaxis is presented in Table 3. Latency duration after PPROM was reduced in the combinations group compared with the 3GC group (Tables 2 and 3, Figure S1). Altogether, 19 perinatal deaths were associated with an infection (*n* = 14/345, *n* = 1/30, *n* = 0/45 and *n* = 4/72 in the amoxicillin, macrolide, 3GC and combinations groups, respectively). Overall, there was no difference in survival and severe neonatal morbidities (Tables 2 and 3). However, differences in survival without severe morbidity were observed (78.5% with amoxicillin, 83.9% with macrolides, 93.6% with 3GC and 86.0% with combinations). After adjusting for gestational age at PPROM and type of unit, the use of a 3GC or a combination was associated with improved survival without severe neonatal morbidity (aRR 1.25, 95% CI 1.08–1.45 and aRR 1.10, 95% CI 1.01–1.20, respectively) when compared with amoxicillin, but not when compared with macrolides (Table 3).

**TABLE 1:** Maternal, obstetric, neonatal and unit characteristics by antibiotic prophylaxis after PPROM

Characteristics	Amoxicillin (*n* = 345)	Macrolide^a^ (*n* = 30)	3GC (*n* = 45)	Combinations^b^ (*n* = 72)	Global *P*‐value
	*n* (%)	*n* (%)	*n* (%)	*n* (%)	
Maternal characteristics					
Age (years) (*n* = 492)					
<20	15 (3.4)	3 (10.3)	5 (9.4)	2 (2.5)	0.09
20–35	260 (77.0)	22 (73.5)	31 (74.2)	61 (87.5)	
>35	70 (19.6)	5 (16.2)	9 (16.4)	9 (10.0)	
Born in France or Europe (*n* = 478)	258 (77.6)	27 (92.9)	36 (74.8)	51 (70.3)	0.31
Married or living with a partner (*n* = 480)	291 (89.1)	25 (89.0)	39 (88.6)	67 (95.0)	0.43
Parents’ socio‐economic position (*n* = 458)					
Manager	71 (20.3)	5 (17.7)	5 (10.9)	10 (12.1)	0.32
Professional	37 (12.9)	4 (13.3)	9 (19.6)	18 (27.1)	
Intermediate^c^	97 (30.2)	11 (37.1)	14 (39.0)	20 (31.1)	
Sales and services worker	52 (17.7)	5 (17.7)	6 (13.2)	9 (16.3)	
Manual worker	45 (14.6)	4 (14.2)	3 (6.9)	8 (9.8)	
Unknown occupation	17 (4.3)	0 (0.0)	5 (10.4)	3 (3.6)	
Primiparity (*n* = 492)	132 (40.4)	16 (53.0)	24 (53.0)	38 (54.3)	0.16
Obstetric characteristics					
PPROM occurring during hospitalisation for another reason (*n* = 492)	51 (12.3)	1 (3.4)	8 (14.4)	5 (6.0)	0.19
Gestational age at PPROM (w) (*n* = 492), median (IQR)	29.0 (26.4–30.9)	29.1 (27.0–30.3)	29.7 (26.3–30.4)	29.3 (26.7–30.7)	0.97
Gestational age at PPROM (w) (*n* = 492)					
24–26	128 (29.0)	7 (21.3)	19 (31.6)	27 (27.7)	0.58
27–29	122 (32.6)	13 (44.5)	10 (19.9)	19 (29.1)	
30–31	95 (38.4)	10 (34.2)	16 (48.5)	26 (43.2)	
Positive vaginal swab at admission (*n* = 375)	111 (40.9)	9 (42.2)	16 (35.4)	22 (43.2)	0.92
Oligohydramnios (*n *= 441)	147 (43.3)	13 (44.9)	21 (40.0)	34 (56.0)	0.38
Gestational age at birth (w) (*n* = 492), median (IQR)	30.6 (28.4–31.9)	29.9 (28.3–31.0)	30.6 (27.1–31.9)	30.6 (28.3–31.9)	0.82
Gestational age at birth (w) (*n* = 492)					
24–26	79 (14.7)	3 (7.6)	12 (17.7)	19 (17.7)	0.49
27–29	101 (25.2)	13 (44.5)	12 (23.8)	21 (26.3)	
30–31	147 (36.7)	14 (47.9)	19 (37.8)	29 (36.4)	
32–34	18 (23.4)	0 (0.0)	2 (20.7)	3 (19.6)	
Obstetric management					
Type 3 maternity unit (with neonatal intensive care unit) (*n* = 492)	321 (87.9)	28 (93.2)	44 (98.0)	68 (89.7)	0.40
In utero transfer (*n* = 492)	219 (61.0)	16 (52.1)	31 (56.5)	59 (79.4)	0.08
Tocolysis (*n* = 492)	271 (79.7)	23 (76.9)	34 (71.8)	60 (85.9)	0.43
Tocolysis duration (*n* = 482)					
No tocolysis	74 (22.0)	7 (23.3)	11 (25.0)	12 (16.7)	0.59
<24 h	56 (16.7)	4 (13.3)	6 (13.6)	18 (25.0)	
24–48 h	126 (37.5)	11 (36.7)	21 (47.8)	28 (38.9)	
>48 h	80 (23.8)	8 (26.7)	6 (13.6)	14 (19.4)	
Antenatal steroids (*n* = 486)					
None	21 (9.0)	3 (10.9)	1 (1.5)	4 (4.4)	0.24
Incomplete course	38 (8.9)	2 (7.3)	4 (6.4)	13 (14.2)	
Complete course	283 (82.1)	23 (81.8)	40 (92.1)	54 (81.3)	
Magnesium sulphate (*n* = 484)	23 (5.6)	2 (7.3)	3 (5.0)	1 (1.3)	0.39
Mode of delivery (*n* = 490)					
Vaginal delivery	152 (43.7)	13 (42.7)	22 (57.4)	34 (44.0)	0.79
Caesarean before labor	135 (40.5)	11 (37.6)	16 (29.2)	26 (37.1)	
Caesarean during labour	57 (15.8)	4 (19.7)	7 (13.4)	11 (19.0)	
Cephalic presentation (*n* = 483)	220 (68.0)	20 (68.1)	37 (85.6)	46 (58.8)	0.06
Neonatal characteristics					
Male fetus (*n* = 492)	187 (51.2)	21 (70.1)	29 (70.2)	37 (53.4)	0.08
Birthweight (g), median (IQR) (*n* = 491)	1450 (1130–1785)	1460 (1180–1580)	1330 (1030–1710)	1420 (1090–1850)	0.45
Small for gestational age (*n* = 491)	66 (20.8)	3 (10.3)	11 (20.8)	10 (11.9)	0.24

Note

All percentages were weighted to account for the sampling design of the EPIPAGE‐2 cohort.

Abbreviations: IQR, interquartile range; w, weeks’ gestation.

^a^ The macrolides group included 17 patients receiving macrolides and 13 patients receiving clindamycin.

^b^ Any combinations of antibiotics covering >90% of *E. coli* in addition to *S. agalactiae*.

^c^ Intermediate socio‐economic position included employees from administration and public services, self‐employed and students.

**TABLE 2:** Outcomes by antibiotic prophylaxis after PPROM

Outcome	Amoxicillin (*n* = 345)	Macrolide (*n* = 30)^a^	3GC (*n* = 45)	Combinations^b^ (*n* = 72)	Global *P*‐value
	*n*/*N* (%)	*n*/*N* (%)	*n*/*N* (%)	*n*/*N* (%)	
Latency duration ≥48 h (*n* = 492)	278/345 (83.2)	24/30 (80.4)	39/45 (89.1)	49/72 (74.4)	0.18
Latency duration ≥7 days (*n* = 492)	128/345 (42.5)	10/30 (33.3)	15/45 (44.0)	19/72 (34.1)	0.64
Intrauterine infection (*n* = 487)	28/340 (6.6)	3/30 (10.3)	2/45 (4.0)	3/72 (3.4)	0.49
Vital status (*n* = 492)					
Termination of pregnancy	1/345 (0.2)	0/30 (0.0)	0/45 (0.0)	0/72 (0.0)	0.95
Antepartum stillbirth	5/345 (0.9)	0/30 (0.0)	0/45 (0.0)	0/72 (0.0)	
Per partum stillbirth	4/345 (0.7)	0/30 (0.0)	0/45 (0.0)	1/72 (0.9)	
Death in delivery room	8/345 (1.6)	1/30 (2.5)	0/45 (0.0)	3/72 (2.8)	
Death in NICU	21/345 (4.3)	2/30 (5.1)	4/45 (5.9)	4/72 (3.7)	
Discharged alive	306/345 (92.3)	27/30 (92.4)	41/45 (94.1)	64/72 (92.5)	
Severe neonatal morbidity					
Early‐onset sepsis (*n* = 453)^c^	15/319 (3.7)	1/28 (3.6)	1/43 (2.1)	0/63 (0.0)	0.43
Late‐onset sepsis (*n* = 453)^c^	51/313 (11.8)	4/29 (14.0)	7/45 (11.9)	9/66 (10.3)	0.96
Any sepsis (*n* = 442)^c^	63/308 (15.2)	5/28 (18.2)	8/44 (14.1)	9/62 (11.6)	0.83
Necrotising enterocolitis (*n* = 465)^c^	12/324 (4.0)	0/29 (0.0)	0/44 (0.0)	2/68 (2.3)	0.47
Severe cerebral lesion (*n* = 458)^c^	24/316 (7.0)	2/29 (6.1)	2/45 (3.0)	3/68 (3.2)	0.43
Severe bronchopulmonary dysplasia (*n* = 414)^d^	19/287 (4.7)	0/25 (0.0)	0/39 (0.0)	2/63 (2.4)	0.30
Retinopathy of prematurity (*n* = 469)^c^	3/327 (0.6)	1/29 (3.5)	0/45 (0.0)	0/72 (0.0)	0.30
Survival at discharge (*n* = 492)	306/345 (92.3)	27/30 (92.4)	41/45 (94.1)	64/72 (92.5)	0.97
Survival at discharge without severe neonatal morbidity^e^ (*n* = 464)	242/325 (78.5)	22/27 (83.9)	37/41 (93.6)	58/71 (86.0)	**0.04**
Survival at 2 yo without neurosensory impairment^f^ (among all fetuses, *n* = 383)	213/266 (83.9)	18/22 (84.8)	31/37 (89.5)	49/58 (88.8)	0.61
Total ASQ score med (IQR) (*n* = 242)	236 (205–265)	215 (200–250)	216 (175–240)	245 (225–280)	0.42
ASQ below threshold (*n* = 242)	69/166 (43.5)	11/15 (73.3)	9/24 (46.7)	14/37 (30.5)	0.13

Note

All percentages were weighted to account for the sampling design of the EPIPAGE‐2 cohort. Indications for termination of pregnancy: PPROM and intrauterine infection at 24 weeks. Causes of stillbirths: PPROM and intrauterine infection (*n* = 4), PPROM and placental abruption (*n* = 1), PPROM and cord prolapse (*n* = 1), unknown (*n* = 4). Causes of death in delivery room: infection and extremely preterm birth (*n* = 8), head entrapment and extremely preterm birth (*n* = 1), extremely preterm birth (*n* = 3). Causes of death in NICU: respiratory distress syndrome (*n* = 10), NEC (*n* = 1, in the Amoxicillin group), sepsis (*n* = 7), central nervous system injury (*n* = 6), other (*n* = 4), unknown (*n* = 3). *P*‐values in bold are statistically significant.

Abbreviations: ASQ, Ages and Stages Questionnaire; IQR, interquartile range; NICU, neonatal intensive care unit.

^a^ The macrolide group included 17 patients receiving macrolides and 13 patients receiving clindamycin.

^b^ Any combinations of antibiotics covering >90% of *E. coli* in addition to *S. agalactiae*.

^c^ Among 469 infants admitted to NICU.

^d^ Among 439 infants alive at 36 weeks.

^e^ Survival at discharge without any of the following: grades III–IV intraventricular haemorrhage, cystic periventricular leucomalacia, stages II–III NEC according to Bell’s staging, stage 3 or greater retinopathy of prematurity or severe bronchopulmonary dysplasia.

^f^ Survival at 2 years of corrected age without cerebral palsy GMFCS levels 2–5 or deafness or blindness.

**TABLE 3:** Association between antibiotic prophylaxis after PPROM and outcomes

Outcome	Cephalosporin vs. Amoxicillin (ref)	Amoxicillin vs. Macrolide (ref)	Cephalosporin vs. Macrolide (ref)
	aRR (95% CI)	aRR (95% CI)	aRR (95% CI)
Latency prolonged by ≥48 h (*n* = 492)^a^	1.07 (0.97–1.19)	1.01 (0.86–1.19)	1.09 (0.92–1.29)
Latency prolonged by ≥7 days (*n* = 492)^a^	0.93 (0.64–1.36)	0.99 (0.62–1.57)	0.92 (0.51–1.65)
Intra‐uterine infection			
CC (*n* = 487)	0.54 (0.15–1.95)	0.99 (0.34–2.86)	0.53 (0.11–2.54)
MI (*n* = 492)	0.53 (0.15–1.93)	1.00 (0.35–2.90)	0.53 (0.11–2.54)
Early‐onset sepsis (among infants admitted to NICU)			
CC (*n* = 390)	0.54 (0.07–4.17)	1.16 (0.16–8.62)	0.62 (0.04–9.42)
MI (*n* = 401)	0.56 (0.07–4.18)	0.98 (0.14–6.82)	0.54 (0.04–7.85)
Late‐onset sepsis (among infants admitted to NICU)			
CC (*n* = 453)	0.76 (0.42–1.37)	1.26 (0.66–2.39)	0.96 (0.46–2.01)
MI (*n* = 469)	0.81 (0.44–1.49)	1.16 (0.61–2.22)	0.94 (0.44–2.02)
Survival at discharge (*n* = 492)^a^	1.04 (0.93–1.16)	1.00 (0.93–1.07)	1.04 (0.92–1.17)
Survival without severe morbidity^b^			
CC (*n* = 464)	**1.26 (1.09–1.45)**	0.90 (0.75–1.08)	1.13 (0.93–1.37)
MI (*n* = 492)	**1.25 (1.08–1.45)**	0.91 (0.76–1.09)	1.14 (0.94–1.39)
Survival at 2 years without neurosensory impairment among all fetuses^c^			
CC (*n* = 383)	1.13 (0.97–1.31)	0.98 (0.86–1.12)	1.10 (0.93–1.31)
MI (*n* = 492)	1.08 (0.94–1.23)	1.00 (0.87–1.14)	1.07 (0.91–1.27)
ASQ below threshold among infants alive at 2 years without neurosensory impairment			
CC (*n* = 242)	0.88 (0.54–1.42)	**0.61 (0.42–0.90)**	**0.54 (0.30–0.96)**
MI (*n* = 420)	0.84 (0.51–1.37)	0.77 (0.51–1.14)	0.64 (0.35–1.18)

	Combinations^d^ vs. Amoxicillin (ref)	Combinations^d^ vs. Macrolide (ref)	Combinations^d^ vs. Cephalosporin (ref)
	aRR (95% CI)	aRR (95% CI)	aRR (95% CI)
Latency prolonged by ≥48 h (*n* = 492)^a^	0.87 (0.72–1.05)	0.88 (0.69–1.11)	**0.81 (0.66–0.99)**
Latency prolonged by ≥7 days (*n* = 492)^a^	0.78 (0.49–1.22)	0.77 (0.40–1.45)	0.83 (0.48–1.44)
Intrauterine infection			
CC (*n* = 487)	0.51 (0.18–1.41)	0.50 (0.12–2.03)	0.94 (0.17–5.10)
MI (*n* = 492)	0.50 (0.18–1.39)	0.50 (0.12–2.03)	0.94 (0.17–5.11)
Late‐onset sepsis (among infants admitted to NICU)			
CC (*n* = 453)	0.88 (0.55–1.39)	1.10 (0.53–2.27)	1.15 (0.59–2.25)
MI (*n* = 469)	0.85 (0.52–1.41)	0.99 (0.48–2.07)	1.06 (0.53–2.12)
Survival at discharge (*n* = 492)^a^	1.00 (0.94–1.07)	1.00 (0.91–1.10)	0.96 (0.87–1.08)
Survival without severe morbidity^b^			
CC (*n* = 464)	**1.11 (1.01–1.21)**	1.00 (0.84–1.18)	0.88 (0.77–1.01)
MI (*n* = 492)	**1.10 (1.01–1.20)**	1.00 (0.85–1.18)	0.88 (0.76–1.01)
Survival at 2 years without neurosensory impairment among all fetuses^c^			
CC (*n* = 383)	1.07 (0.97–1.17)	1.04 (0.89–1.21)	0.94 (0.81–1.11)
MI (*n* = 492)	1.04 (0.95–1.14)	1.04 (0.89–1.22)	0.97 (0.84–1.12)
ASQ below threshold among infants alive at 2 years without neurosensory impairment			
CC (*n* = 242)	0.94 (0.57–1.55)	**0.58 (0.34–0.98)**	1.07 (0.58–1.97)
MI (*n* = 420)	0.97 (0.63–1.48)	0.74 (0.44–1.24)	1.15 (0.65–2.04)

Note

Adjusted risk ratios (aRR) obtained from population‐averaged Poisson regression models with robust variance estimation, adjusted for gestational age at PPROM and the type of maternity unit (except for intrauterine infection adjusted only for gestational age at PPROM, as there were no intrauterine infections diagnosed with delivery in type 1 maternity units). There was no case of early‐onset sepsis in the combination group, hence this outcome is not reported in the second part of the table. *P*‐values in bold are statistically significant.

Abbreviations: aRR, adjusted risk ratios; ASQ, Ages and Stages Questionnaire; CC, complete cases; MI, multiple imputation; ref, reference.

^a^ No missing data.

^b^ Survival at discharge without any of the following: grades III–IV intraventricular haemorrhage, cystic periventricular leucomalacia, stages II–III NEC according to Bell’s staging, stage 3 or greater retinopathy of prematurity or severe bronchopulmonary dysplasia.

^c^ Survival at 2 years of corrected age without cerebral palsy GMFCS levels 2–5 or deafness or blindness.

^d^ Any combinations of antibiotics covering >90% of *E. coli* in addition to *S. agalactiae*.

With antibiotic prophylaxis using amoxicillin, a macrolide, a 3GC or an *E. coli*‐targeting combination, 83.9%, 84.8%, 89.5% and 88.8% of exposed fetuses were alive at 2 years’ corrected age without neurosensory impairment, respectively. Among the infants with a neurodevelopmental screening questionnaire between 22 and 26 months, 43.5%, 73.3%, 46.7% and 30.5% had an ASQ score below threshold (Table 2). Multivariate analyses did not reveal any association between antibiotic prophylaxis and neurosensory impairment. However, macrolides were associated with poorer neurodevelopmental outcome compared with the other groups in the complete cases analysis, but this association disappeared after multiple imputations (Table 3). A sensitivity analysis after stratification on gestational age at PPROM showed consistent results (Table S4).

We further investigated the episodes of neonatal sepsis. There were 17 cases of EOS, 15 in the amoxicillin group (10/15 caused by *E. coli*, of which six were ampicillin‐resistant and one was resistant to both ampicillin and 3GC, and 1/15 caused by ampicillin‐resistant *S. agalactiae*), one in the macrolide group (caused by *Streptococcus agalactiae*), one in the 3GC group (caused by *Staphylococcus epidermidis*) and none in the combinations group. Seventy‐one infants had a total of 98 episodes of LOS, 51 infants in the amoxicillin group (69 LOS), four in the macrolide group (four LOS), seven in the 3GC group (10 LOS) and nine in the combinations group (15 LOS). The most common pathogens in blood cultures collected during LOS were Gram‐positive organisms (coagulase negative staphylococci [53/98], *Staphylococcus* *aureus* [11/98], *Enterococcus* sp. [2/98] or another non‐specified pathogen [8/98]), followed by Gram‐negative organisms (*E*. *coli* [8/98], *Acinetobacter baumanii* [2/98], *Enterobacter cloacae* [3/98] or another pathogen [9/98]) and *Candida* sp. (2/98). LOS involved a pathogen resistant to the initial antibiotic prophylaxis regimen in 24 neonates: 21 from the amoxicillin group, one from the 3GC group and two from the *E*. *coli*‐ targeting combinations group.

## 4 DISCUSSION

### 4.1 Main findings

After PPROM at 24–31 weeks, antibiotic prophylaxis based on 3GC was associated with higher survival without severe neonatal morbidity than amoxicillin. To a weaker extent, the same pattern was found when comparing antibiotic combinations covering *S*. *agalactiae* and >90% of *E*. *coli* with amoxicillin. We evidenced no increase of EOS/LOS related to 3GC‐resistant pathogen among infants whose mothers received antenatal 3GC prophylaxis at 24–31 weeks.

### 4.2 Strengths and limitations

Strengths of our study include a population‐based sample of women with PPROM at a national level, with obstetrical management fitting the current clinical guidelines, and detailed high‐quality data covering pregnancy, neonatal hospitalisation (including bacterial documentation) and follow‐up at 2 years. Considering the paucity of evidence‐based data regarding the comparison of different antibiotic prophylaxis, such observational studies are instrumental in assessing routine clinical practices in a real‐life setting. We could evaluate the administration of amoxicillin and 3GC in this indication, which has commonly been overlooked in previous studies,^27^ or has only been studied in combination with other antibiotics, which makes it challenging to determine their intrinsic effect.^28^, ^29^, ^30^

The main limitations of our work should be kept in mind: a limited sample size and the large number of comparisons performed. The observed significant associations are biologically plausible but may be due to chance, although all outcomes were pre‐specified based on clinical relevance and previous studies on the topic.^31^ Due to the right‐truncation of women with PPROM before 34 weeks who delivered from 35 weeks on (i.e. not eligible for the EPIPAGE‐2 cohort), we restricted our analyses to women with PPROM at 24–31 weeks and therefore likely missed a few births with the longest latency durations and best prognoses. Antibiotic treatments were compared irrespective of administration routes, dosages or durations, as these were beyond the scope of this study. As we focused on the initial management strategy implemented upon diagnosis of PPROM, and considering that most women gave birth within the first week after PPROM, we did not explore subsequent antibiotic administrations. Finally, bacterial ecology might have evolved since 2011, in particular with an increase in the frequency of extended‐spectrum beta‐lactamase‐producing bacteria.^32^

### 4.3 Interpretation

The optimal antibiotic prophylaxis in the setting of PPROM should ideally combine good maternal and fetal tolerance profile and narrow spectrum focusing on the likely bacterial pathogens involved, namely, *S*. *agalactiae* and *E*. *coli*. Whether the antimicrobial spectrum should include genital mycoplasmas (*Ureaplasma* and other genital *Mycoplasma* species) remains unresolved: as ordinary colonisers of the lower genital tract, their frequent documentation in histologically confirmed intrauterine infection may be considered as by‐standing colonisation, a viewpoint reinforced by the lack of demonstrated benefit of any *Mycoplasma*‐targeting antibiotic in the management of intrauterine infection.^15^, ^33^ Conversely, some authors have provided evidence for potential pathogenicity in histologically confirmed intrauterine infections, including induction of local inflammatory response with deleterious consequences in animal models.^14^ In this perspective, the optimal antibiotic choice is not straightforward, as all candidates exhibit pros and cons. Amoxicillin/ampicillin and penicillin are effective on 100% of *S*. *agalactiae* isolates but on no more than 60% of *E. coli* isolates, according to the European Committee on Antimicrobial Susceptibility Testing (EUCAST) data.^34^ Erythromycin, spiramycin and clindamycin are active on 75–80% of *S*. *agalactiae* but exhibit only marginal activity toward *E*. *coli*.^34^ In addition, macrolides have limited transplacental passage,^35^ which could limit the prevention of materno‐fetal infection.^27^ 3GC exhibit a broader spectrum that, beyond *S*. *agalactiae*, encompasses >90% of *E*. *coli* isolates, according to EUCAST data.^34^ On the other hand, 3GC remain ineffective towards intracellular *Mycoplasma* sp., raising more concerns regarding the potential greater impact on maternal/neonatal gut microbiota.^16^, ^28^ Oral 3GC, because of their poor oral bioavailability, are not recommended in this setting.^36^

In the present study, we could not evidence any superiority of amoxicillin, macrolides, 3GC or any combinations over the others regarding the reduction of intrauterine infection and neonatal sepsis. However, our results suggest a trend towards an overall beneficial effect of 3GC administration, with improved infant survival at discharge without severe neonatal morbidity. This was also found in the combinations group, which antibacterial spectrum could be considered roughly similar to the 3GC regimen, except for the subsets with additional anaerobic and mycoplasma coverage provided by clindamycin and macrolides, respectively. These differences did not translate into statistical differences in survival at 2 years without neurosensory impairment. This should be confirmed in a further study by studying complementary outcomes (e.g. minor morbidity) in the longer term, as some differences might become apparent later in life (as was the case in the ORACLE II trial^37^).

We collected data regarding bacterial documentation in EOS and LOS that confirm the high involvement of *E*. *coli* in EOS and highlight the high frequency of amoxicillin‐resistance among *E*. *coli* isolates collected in infants after maternal exposure to amoxicillin. The latter observation is in line with previous publications that evidenced an increase in ampicillin‐resistant strains infections in preterm neonates whose mothers had received antenatal ampicillin, including in the setting of PPROM prophylaxis.^38^, ^39^, ^40^

Our findings also suggested poorer neurodevelopment at 2 years’ corrected age in infants exposed to antenatal macrolides. This result should be interpreted very cautiously as (i) few women received this treatment, (ii) the trend was similar but no longer significant after multiple imputations, (iii) other factors, not taken into account in the analyses, could be associated with neurodevelopmental outcomes, and (iv) ASQ was estimated through a parental questionnaire, which results are to be confirmed based on the cognitive assessment by a neuropsychologist at the 5‐year follow‐up. No long‐term effect of erythromycin was identified in the ORACLE I trial (the largest randomised controlled trial comparing different broad‐spectrum antibiotics among 4826 women with PPROM).^41^ However, in the ORACLE II trial (comparing antibiotics in women with spontaneous preterm labour and intact membranes), erythromycin was associated with an increase in functional impairment and cerebral palsy among children at 7 years of age.^37^

### 4.4 Clinical and research implications

Whether the overall beneficial effect of 3GC or combination regimens could be attributed to the extended *E*. *coli* coverage and subsequent reduction of infection burden and related morbi‐mortalities is likely but should be further studied. Although no deleterious effect of neither 3GC or combinations treatments could be evidenced in this study, theoretical concerns remain regarding the impact of such broad spectrum antibiotics (3GC) or combinations on maternal and neonatal gut microbiota.^42^, ^43^ This should, however, be considered with the fact that most preterm infants born after PPROM will be exposed to antibiotics after birth.^44^, ^45^ It has also been recently emphasised that the clinical spectrum of one given antibiotic does not translate into ecological impact on the microbiota, the later depending on multiple factors including concentrations achieved in the colon.^46^ Altogether, given these reservations and the lack of any additional benefit evidenced for combinations, at present such combined therapies should be discarded.

Finally, these exploratory findings based on observational data are not intended to lead to a change in clinical practice. The observed associations generate hypotheses that need to be confirmed or refuted in a subsequent study with a larger sample and pre‐planned hypotheses. The impact on maternal and neonatal microbiota, and resistances, optimal posology and duration of antibiotic prophylaxis remain to be determined.

## 5 CONCLUSION

Optimisation of the use of antibiotics by using the more restricted antibiotic spectrum with the most favourable clinical outcome, is a global priority to prevent antimicrobial resistance and preserve the efficacy of existing antibiotics. This is true as well for preterm babies, before and after birth. Evidence‐based data arising from new well‐conducted studies and randomised controlled trials, with long‐term follow‐up, are needed to better define which antibiotics are to be preferentially used after PPROM, and their modalities of administration.

## CONFLICT OF INTERESTS

None declared. Completed disclosure of interest forms are available to view online as supporting information.

## AUTHOR CONTRIBUTIONS

Study concept and design of the present study: EL, CC, GK. Acquisition, analysis and interpretation of data: EL, ML, HT, LFLH, CG‐LG, VB, PB, CC, GK. Statistical analysis: EL. Drafting of the manuscript: EL, CC, GK. Critical revision of the manuscript for important intellectual content: EL, ML, HT, LFLH, CG‐LG, VB, PB, CC, GK. Final approval of the version to be published: EL, ML, HT, LFLH, CG‐LG, VB, PB, CC, GK. Agreement to be accountable for all aspects of the work: EL, ML, HT, LFLH, CG‐LG, VB, PB, CC, GK.

## ETHICS APPROVAL

Recruitment in the EPIPAGE‐2 cohort study and follow‐up evaluations were approved by the appropriate ethics committees, i.e. the advisory committee on the treatment of personal health data for research purposes (references 10‐626, 12‐109 and 16‐263) and the committee for the protection of people participating in biomedical research (references 2011‐A00159‐32 and 2016‐A0033‐48). All the procedures are in accordance with the Declaration of Helsinki of the World Medical Association.

## Supporting information

Fig S1

Table S1

Table S2

Table S3

Table S4

Supplementary Material

Supplementary Material

Supplementary Material

Supplementary Material

Supplementary Material

Supplementary Material

Supplementary Material

Supplementary Material

Supplementary Material

## Data Availability

The data that support the findings of this study are available from the corresponding author upon reasonable request.
