# Supplementary material for: Antibiotic prophylaxis in preterm premature rupture of membranes at 24–31 weeks’ gestation: Perinatal and 2‐year outcomes in the EPIPAGE‐2 cohort
Source: BJOG. 2022 Jan 13;129(9):1560–73. doi: 10.1111/1471-0528.17081 (PMC9546066; doi:10.1111/1471-0528.17081)
Supplement: Supplementary file 2 — Table S1 [file BJO-129-1560-s003.docx]

Table S1: Comparison of characteristics and outcomes between the study population and two excluded groups (women with an alternative antibiotic regimen and women with no antibiotic after preterm premature rupture of membranes)

|  | | | **Included** | **Excluded** | |  |
| --- | --- | --- | --- | --- | --- | --- |
| **Characteristics and outcomes** | | | **Study population (n=492)** | **Alternative antibiotic regimen (n=60)** | **No antibiotic after PPROM (n=41)** | **Global P value** |
| **Maternal characteristics** | | |  |  |  |  |
| Age (years) (n=593) | | |  |  |  | .89 |
|  | <20 | | 25/492 (4.2) | 3/60 (4.0) | 2/41 (0.4) |  |
|  | 20-35 | | 37/492 (78.0) | 50/60 (80.8) | 31/41 (72.0) |  |
|  | >35 | | 93/492 (17.8) | 7/60 (15.2) | 8/41 (24.3) |  |
| Born in France or Europe (n=578) | | | 372/478 (77.1) | 44/59 (73.8) | 37/41 (91.5) | .16 |
| Married or living with a partner (n=580) | | | 422/480 (89.9) | 52/60 (88.7) | 36/40 (91.9) | .86 |
| Parents’ socioeconomic position (n=553) | | |  |  |  | .15 |
|  | Manager | | 91/458 (18.1) | 14/57 (20.1) | 7/38 (14.6) |  |
|  | Professional | | 68/458 (15.6) | 10/57 (14.1) | 9/38 (39.0) |  |
|  | Intermediate | | 142/458 (31.4) | 19/57 (40.5) | 12/38 (25.8) |  |
|  | Sales and services worker | | 72/458 (17.2) | 5/57 (13.2) | 5/38 (10.6) |  |
|  | Manual worker | | 60/458 (13.2) | 9/57 (12.1) | 4/38 (7.8) |  |
|  | Unknown occupation | | 25/458 (4.5) | 0/57 (0.0) | 1/38 (2.2) |  |
| Primiparity (n=592) | | | 210/492 (44.2) | 28/60 (49.5) | 20/40 (40.6) | .75 |
| **Obstetric characteristics** | | |  |  |  |  |
| PPROM occurring during hospitalization for another reason (n=593) | | | 65/492 (11.1) | 11/60 (15.3) | 9/41 (18.0) | .31 |
| Gestational age at PPROM (w) (n=593) | | |  |  |  | .12 |
|  | 24-26 | | 181/492 (28.7) | 24/60 (29.0) | 11/41 (18.4) |  |
|  | 27-29 | | 164/492 (31.5) | 23/60 (33.6) | 18/41 (56.1) |  |
|  | 30-31 | | 147/492 (39.8) | 13/60 (37.4) | 12/41 (25.5) |  |
| Gestational age at birth (w) (n=593) | | |  |  |  | .86 |
|  | 24-26 | | 113/492 (15.0) | 16/60 (17.4) | 9/41 (14.2) |  |
|  | 27-29 | | 147/492 (26.3) | 23/60 (33.6) | 14/41 (29.7) |  |
|  | 30-31 | | 209/492 (37.3) | 18/60 (26.2) | 16/41 (34.0) |  |
|  | 32-34 | | 23/492 (21.4) | 3/60 (22.8) | 2/41 (22.1) |  |
| **Obstetric management** | | |  |  |  |  |
| Type 3 maternity unit (n=593) | | | 461/492 (89.3) | 56/60 (88.8) | 32/41 (74.2) | .21 |
| *In utero* transfer (n=593) | | | 325/492 (62.8) | 32/60 (51.0) | 26/41 (61.4) | .40 |
| Tocolysis (n=592) | | | 388/492 (79.7) | 41/60 (68.8) | 22/40 (54.3) | .009 |
| Antenatal steroids (n=585) | | |  |  |  | <.001 |
|  | None | | 29/486 (7.7) | 5/59 (12.4) | 16/40 (32.5) |  |
|  | Incomplete course | | 57/486 (9.4) | 4/59 (5.5) | 12/40 (23.8) |  |
|  | Complete course | | 400/486 (82.9) | 50/59 (82.0) | 12/40 (43.7) |  |
| Magnesium sulfate (n=583) | | | 29/484 (5.0) | 3/59 (4.1) | 3/40 (6.0) | .90 |
| Mode of delivery (n=589) | | |  |  |  | .36 |
|  | Vaginal delivery | | 221/490 (44.9) | 31/60 (47.6) | 25/39 (61.9) |  |
|  | Cesarean before labor | | 188/490 (38.8) | 23/60 (44.0) | 11/39 (22.7) |  |
|  | Cesarean during labor | | 81/490 (16.3) | 6/60 (8.4) | 3/39 (15.4) |  |
| **Neonatal characteristics** | | |  |  |  |  |
| Male fetus (n=593) | | | 274/492 (54.2) | 28/60 (42.9) | 28/41 (65.1) | .23 |
| Birthweight (grams), median (IQR) (n=592) | | | 1425 (1120-1770) | 1300 (982-1644) | 1450 (1080-2155) | .19 |
| Small for gestational age (n=592) | | | 90/491 (18.9) | 11/60 (21.4) | 5/41 (10.1) | .44 |
| **Prolongation of gestation** | | |  |  |  |  |
| Latency duration (n=585) median (IQR) | | | 5.1 (2.5-12.0) | 6.0 (2.2-10.2) | 3.0 (0.4-30.0) | .10 |
| Latency prolonged by ≥ 48 hr (n=592) | | | 390/492 (82.4) | 44/59 (79.4) | 19/41 (56.6) | .002 |
| Latency prolonged by ≥ 7 days (n=592) | | | 172/492 (40.9) | 21/59 (40.7) | 11/41 (40.7) | .99 |
| **Intra-uterine infection** (n=586) | | | 36/487 (6.1) | 6/59 (14.3) | 1/40 (2.2) | .12 |
| **Neonatal outcomes** | | |  |  |  |  |
| Vital status (n=593) | | |  |  |  | .05 |
|  | | Termination of pregnancy | 1/492 (0.1) | 0/60 (0) | 1/41 (2.1) |  |
|  | | Antepartum stillbirth | 5/492 (0.7) | 0/60 (0) | 0/41 (0) |  |
|  | | Per-partum stillbirth | 5/492 (0.7) | 3/60 (3.2) | 2/41 (3.7) |  |
|  | | Death in delivery room | 12/492 (1.6) | 1/60 (1.1) | 2/41 (3.7) |  |
|  | | Death in NICU | 31/492 (4.4) | 7/60 (9.1) | 2/41 (4.3) |  |
|  | | Discharged alive | 438/492 (92.5) | 49/60 (86.6) | 34/41 (86.2) |  |
| **Severe neonatal morbidity** | | |  |  |  |  |
|  | | Early-onset sepsis (n=542)^a^ | 17/453 (3.1) | 3/54 (4.2) | 2/35 (4.8) | .76 |
|  | | Late-onset sepsis (n=542)^a^ | 71/453 (11.7) | 7/53 (9.9) | 7/36 (14.6) | .77 |
|  | | Any sepsis (n=529)^a^ | 85/442 (14.8) | 9/51 (14.1) | 9/36 (19.3) | .74 |
|  | | Necrotizing enterocolitis (n=557)^a^ | 14/465 (3.2) | 0/56 (0.0) | 1/36 (2.4) | .48 |
|  | | Severe cerebral lesion (n=547)^a^ | 31/458 (6.0) | 4/55 (6.2) | 5/34 (12.3) | .34 |
|  | | Severe bronchopulmonary dysplasia (n=491) | 21/413 (3.7) | 1/46 (1.3) | 1/32 (1.9) | .47 |
|  | | Retinopathy of prematurity (n=561)^a^ | 4/469 (0.6) | 0/56 (0) | 0/36 (0) | .75 |
| Survival at discharge (n=593) | | | 438/492 (92.5) | 49/60 (86.6) | 34/41 (86.2) | .12 |
| Survival without severe morbidity (n=557)^c^ | | | 359/464 (81.2) | 43/56 (82.0) | 25/37 (72.6) | .45 |
| Survival at 2yo without neurosensory impairment (among all fetuses, n=463)^d^ | | | 311/383 (85.2) | 36/47 (80.8) | 24/33 (72.8) | .16 |
| Total ASQ score median (IQR) (n=283) | | | 235 (252-264) | 240 (220-255) | 195 (178-245) | .66 |
| ASQ below threshold (n=283) | | | 103/242 (43.4) | 8/25 (27.7) | 7/16 (57.1) | .26 |

ASQ: Ages and Stages Questionnaire, IQR: interquartile range, NICU: neonatal intensive care unit, PPROM: preterm premature rupture of membranes, w: weeks’ gestation

^a^ Among 561 infants admitted to NICU

^b^ Among 521 infants alive at 36 weeks

^c^ Survival at discharge without any of the following: grades III-IV intraventricular hemorrhage, cystic periventricular leukomalacia, stages II-III NEC according to Bell’s staging, stage 3 or greater retinopathy of prematurity or severe bronchopulmonary dysplasia.

^d^ Survival at 2 years of corrected age without cerebral palsy GMFCS levels 2-5 or deafness or blindness
