# Supplementary material for: Antibiotic prophylaxis in preterm premature rupture of membranes at 24–31 weeks’ gestation: Perinatal and 2‐year outcomes in the EPIPAGE‐2 cohort
Source: BJOG. 2022 Jan 13;129(9):1560–73. doi: 10.1111/1471-0528.17081 (PMC9546066; doi:10.1111/1471-0528.17081)
Supplement: Supplementary file 3 — Table S2 [file BJO-129-1560-s002.docx]

Table S2: Antibiotic prophylaxis regimens

| Antibiotic prophylaxis regimens | | n |
| --- | --- | --- |
| Penicillins | |  |
|  | **Amoxicillin** | **345** |
|  | Co-amoxiclav | 19 |
|  | Other penicillin (penicillin A) | 1 |
| Third-generation cephalosporins | |  |
|  | **Ceftriaxone** | **32** |
|  | **Cefotaxime** | **13** |
|  | Cefixime | 5 |
| First-generation cephalosporins (cefazolin) | | 8 |
| Macrolides and related | |  |
|  | **Erythromycin** | **16** |
|  | **Clindamycin** | **13** |
|  | **Spiramycin** | **1** |
| Combinations | |  |
|  | Amoxicillin + Macrolide | 21 |
|  | **Amoxicillin + 3GC** | **19** |
|  | Amoxicillin + First-generation cephalosporin | 1 |
|  | Amoxicillin + Metronidazole | 3 |
|  | **Amoxicillin + Aminoglycoside** | **6** |
|  | **Other β-lactam + 3GC** | **2** |
|  | **Other β-lactam + Aminoglycoside** | **3** |
|  | **3GC + First-generation cephalosporin** | **1** |
|  | **Macrolide + 3GC** | **1** |
|  | Macrolide + Clindamycin | 2 |
|  | **3GC + Aminoglycoside** | **1** |
|  | **3GC + Metronidazole** | **7** |
|  | **Amoxicillin + 3GC + Macrolide** | **2** |
|  | **Amoxicillin + 3GC + Aminoglycoside** | **6** |
|  | **Amoxicillin + 3GC + Metronidazole** | **1** |
|  | **3GC + Aminoglycoside + Metronidazole** | **18** |
|  | **3GC + Macrolide + Metronidazole** | **1** |
|  | **Amoxicillin + Metronidazole + Aminoglycoside** | **1** |
|  | **Amoxicillin + 3GC + Aminoglycoside + Metronidazole** | **2** |
|  | **3GC + Clindamycin + Aminoglycoside + Metronidazole** | **1** |

In bold, antibiotic prophylaxis regimens analyzed in this study.
