# Supplementary material for: Antibiotic prophylaxis in preterm premature rupture of membranes at 24–31 weeks’ gestation: Perinatal and 2‐year outcomes in the EPIPAGE‐2 cohort
Source: BJOG. 2022 Jan 13;129(9):1560–73. doi: 10.1111/1471-0528.17081 (PMC9546066; doi:10.1111/1471-0528.17081)
Supplement: Supplementary file 4 — Table S3 [file BJO-129-1560-s011.docx]

Table S3: Comparison of characteristics of participants with and without follow-up among the 434 children alive at 2 years’ corrected age

|  | | | **With follow-up at 2 years (n=366)** | **Without follow-up at 2 years (n=68)** | **p-value** |
| --- | --- | --- | --- | --- | --- |
| **Maternal characteristics at birth** | | |  |  |  |
| Age (years) (n=434) | | |  |  | **.03** |
|  | <20 | | 12/366 (2.7) | 10/68 (10.9) |  |
|  | 20-35 | | 285/366 (79.9) | 48/68 (72.7) |  |
|  | >35 | | 69/366 (17.4) | 10/68 (16.4) |  |
| Born in France or Europe (n=425) | | | 280/360 (76.7) | 50/65 (78.1) | .85 |
| Married or living with a partner (n=422) | | | 313/359 (89.4) | 55/63 (90.5) | .78 |
| Parents’ socioeconomic position (n=407) | | |  |  | .79 |
|  | Manager | | 76/347 (19.2) | 7/60 (14.1) |  |
|  | Professional | | 53/347 (15.7) | 10/60 (17.9) |  |
|  | Intermediate | | 107/347 (31.3) | 15/60 (28.3) |  |
|  | Sales and services worker | | 57/347 (17.8) | 9/60 (16.7) |  |
|  | Manual worker | | 40/347 (12.7) | 13/60 (15.4) |  |
|  | Unknown occupation | | 14/347 (3.3) | 6/60 (7.6) |  |
| Primiparity (n=434) | | | 152/366 (43.9) | 36/68 (45.4) | .86 |
| **Obstetric characteristics** | | |  |  |  |
| PPROM occurring during hospitalization for another reason (n=434) | | | 47/366 (11.0) | 7/68 (7.8) | .40 |
| Gestational age at PPROM (w) (n=434) | | |  |  | .11 |
|  | 24-26 | | 106/366 (23.5) | 20/68 (20.4) |  |
|  | 27-29 | | 133/366 (31.1) | 28/68 (46.8) |  |
|  | 30-31 | | 127/366 (45.4) | 20/68 (32.8) |  |
| Gestational age at birth (w) (n=434) | | |  |  | .62 |
|  | 24-26 | | 54/366 (9.4) | 9/68 (7.7) |  |
|  | 27-29 | | 118/366 (27.6) | 22/68 (25.4) |  |
|  | 30-31 | | 176/366 (41.1) | 32/68 (36.9) |  |
|  | 32-34 | | 18/366 (21.9) | 5/68 (30.0) |  |
| **Obstetric management** | | |  |  |  |
| Type 3 maternity unit (n=434) | | | 340/366 (87.1) | 66/68 (97.7) | **.007** |
| *In utero* transfer (n=434) | | | 239/366 (62.4) | 46/68 (61.3) | .90 |
| Tocolysis (n=434) | | | 292/366 (80.4) | 52/68 (78.2) | .74 |
| Antenatal steroids (n=429) | | |  |  | **.04** |
|  | None | | 16/362 (7.6) | 4/67 (4.4) |  |
|  | Incomplete course | | 45/362 (10.0) | 2/67 (2.0) |  |
|  | Complete course | | 301/362 (82.4) | 61/67 (93.6) |  |
| Magnesium sulfate (n=427) | | | 23/360 (5.3) | 3/67 (3.5) | .49 |
| Mode of delivery (n=432) | | |  |  | .20 |
|  | Vaginal delivery | | 159/365 (45.5) | 27/67 (34.9) |  |
|  | Cesarean before labor | | 146/365 (39.8) | 26/67 (39.3) |  |
|  | Cesarean during labor | | 60/365 (14.7) | 14/67 (25.8) |  |
| **Neonatal characteristics** | | |  |  |  |
| Male fetus (n=434) | | | 208/366 (55.4) | 34/68 (47.7) | .37 |
| Birthweight (grams), median (IQR) (n=434) | | | 1480 (1200-1800) | 1550 (1128-1785) | .99 |
| Small for gestational age (n=434) | | | 62/366 (18.9) | 14/68 (16.2) | .61 |
| **Prolongation of gestation** | | |  |  |  |
| Latency duration (n=429) median (IQR) | | | 5.6 (2.5-12.0) | 6.5 (3.2-21.2) | .36 |
| Latency prolonged by ≥ 48 hr (n=434) | | | 289/366 (81.7) | 57/68 (88.5) | .15 |
| Latency prolonged by ≥ 7 days (n=434) | | | 134/366 (41.3) | 26/68 (49.1) | .37 |
| **Intra-uterine infection** (n=429) | | | 26/361 (6.0) | 3/68 (3.2) | .29 |
| **Neonatal outcomes** | | |  |  |  |
| Severe neonatal morbidity | | |  |  |  |
|  | | Early-onset sepsis (n=420)^a^ | 11/354 (2.6) | 3/66 (3.2) | .76 |
|  | | Late-onset sepsis (n=426)^a^ | 49/360 (10.3) | 13/66 (13.2) | .44 |
|  | | Any sepsis (n=415)^a^ | 59/351 (13.2) | 14/64 (14.8) | .72 |
|  | | Necrotizing enterocolitis (n=432)^a^ | 10/365 (3.3) | 0/67 (0.0) | .27 |
|  | | Severe cerebral lesion (n=428)^a^ | 11/362 (3.5) | 6/66 (6.9) | .24 |
|  | | Severe bronchopulmonary dysplasia (n=410) | 16/351 (3.3) | 3/59 (4.0) | .76 |
|  | | Retinopathy of prematurity (n=434)^a^ | 3/366 (0.6) | 1/68 (1.2) | .55 |
| Survival without severe morbidity (n=406)^c^ | | | 306/346 (88.7) | 51/60 (88.3) | .93 |

IQR: interquartile range, NICU: neonatal intensive care unit, PPROM: preterm premature rupture of membranes, w: weeks’ gestation

^a^ Among infants admitted to NICU

^b^ Among infants alive at 36 weeks

^c^ Survival at discharge without any of the following: grades III-IV intraventricular hemorrhage, cystic periventricular leukomalacia, stages II-III NEC according to Bell’s staging, stage 3 or greater retinopathy of prematurity or severe bronchopulmonary dysplasia.

^d^ Survival at 2 years of corrected age without cerebral palsy GMFCS levels 2-5 or deafness or blindness
