# Supplementary material for: Antibiotic prophylaxis in preterm premature rupture of membranes at 24–31 weeks’ gestation: Perinatal and 2‐year outcomes in the EPIPAGE‐2 cohort
Source: BJOG. 2022 Jan 13;129(9):1560–73. doi: 10.1111/1471-0528.17081 (PMC9546066; doi:10.1111/1471-0528.17081)
Supplement: Supplementary file 5 — Table S4 [file BJO-129-1560-s005.docx]

Table S4: Association between antibiotic prophylaxis and outcomes after stratification for gestational age at PPROM

| **Outcome** | | **Cephalosporin**  **vs Amoxicillin (ref)** | **Amoxicillin vs Macrolide (ref)** | **Cephalosporin**  **vs Macrolide (ref)** |
| --- | --- | --- | --- | --- |
|  | | aRR (95% CI) | aRR (95% CI) | aRR (95% CI) |
| **Latency prolonged by ≥ 48 hr** | |  |  |  |
|  | 24-26 w (n=181)^a^ | 1.01 (0.86-1.20) | 1.04 (0.82-1.32) | 1.05 (0.81-1.37) |
|  | 27-31 w (n=311)^a^ | 1.10 (0.98-1.24) | 1.00 (0.82-1.22) | 1.10 (0.90-1.34) |
| **Latency prolonged by ≥ 7 days** | |  |  |  |
|  | 24-26 w (n=181)^a^ | 0.91 (0.55-1.51) | 0.72 (0.48-1.09) | 0.66 (0.33-1.33) |
|  | 27-31 w (n=311)^a^ | 0.89 (0.53-1.53) | 1.42 (0.63-3.21) | 1.27 (0.60-2.69) |
| **Survival at discharge** | |  |  |  |
|  | 24-26 w (n=181)^a^ | 1.08 (0.82-1.43) | 1.17 (0.75-1.81) | 1.26 (0.76-2.11) |
|  | 27-31 w (n=311)^a^ | 1.02 (0.99-1.04) | 0.97 (0.94-1.00) | 0.99 (0.97-1.01) |
| **Survival at discharge without severe neonatal morbidity^b^** | | |  |  |
|  | 24-26 w CC (n=170) | **1.51 (1.09-2.07)** | 0.77 (0.52-1.14) | 1.15 (0.72-1.84) |
|  | 24-26 w MI (n=181) | **1.46 (1.07-2.01)** | 0.80 (0.53-1.20) | 1.17 (0.73-1.87) |
|  | 27-31 w CC (n=294) | **1.16 (1.08-1.25)** | 0.94 (0.79-1.12) | 1.09 (0.95-1.26) |
|  | 27-31 w MI (n=311) | **1.16 (1.06-1.26)** | 0.95 (0.79-1.13) | 1.10 (0.93-1.29) |
| **Survival at 2yo without neurosensory impairment among all fetuses^c^** | | | |  |
|  | 24-26 w CC (n=148) | 1.23 (0.85-1.77) | 1.01 (0.61-1.67) | 1.24 (0.75-2.06) |
|  | 24-26 w MI (n=181) | 1.15 (0.82-1.63) | 1.08 (0.61-1.89) | 1.24 (0.69-2.21) |
|  | 27-31 w CC (n=235) | **1.06 (1.01-1.12)** | 0.98 (0.86-1.12) | 1.04 (0.92-1.18) |
|  | 27-31 w MI (n=311) | 1.04 (0.94-1.15) | 1.00 (0.87-1.14) | 1.03 (0.89-1.20) |
| **ASQ below threshold among infants alive at 2yo without neurosensory impairment** | | | |  |
|  | 24-26 w CC (n=72) | 0.50 (0.16-4.15) | 0.60 (0.24-1.48) | 0.30 (0.06-1.48) |
|  | 24-26 w MI (n=119) | 0.79 (0.30-2.08) | 0.66 (0.27-1.61) | 0.52 (0.14-1.96) |
|  | 27-31 w CC (n=170) | 1.16 (0.65-2.08) | **0.58 (0.39-0.87)** | 0.67 (0.36-1.27) |
|  | 27-31 w MI (n=301) | 0.86 (0.48-1.54) | 0.77 (0.51-1.16) | 0.66 (0.33-1.32) |
|  | | **Combination**^d^ **vs Amoxicillin (ref)** | **Combination**^d^ **vs Macrolide (ref)** | **Combination**^d^ **vs Cephalosporin (ref)** |
|  | | aRR (95% CI) | aRR (95% CI) | aRR (95% CI) |
| **Latency prolonged by ≥ 48 hr** | |  |  |  |
|  | 24-26 w (n=181)^a^ | **0.71 (0.53-0.96)** | 0.74 (0.51-1.07) | **0.70 (0.51-0.96)** |
|  | 27-31 w (n=311)^a^ | 0.97 (0.81-1.16) | 0.97 (0.74-1.25) | 0.88 (0.72-1.07) |
| **Latency prolonged by ≥ 7 days** | |  |  |  |
|  | 24-26 w (n=181)^a^ | 0.65 (0.32-1.33) | 0.47 (0.20-1.08) | 0.71 (0.31-1.65) |
|  | 27-31 w (n=311)^a^ | 0.86 (0.42-1.77) | 1.23 (0.43-3.38) | 0.96 (0.43-2.17) |
| **Survival at discharge** | |  |  |  |
|  | 24-26 w (n=181)^a^ | 0.96 (0.76-1.21) | 1.12 (0.68-1.84) | 0.89 (0.64-1.23) |
|  | 27-31 w (n=311)^a^ | **1.02 (1.00-1.04)** | 0.99 (0.97-1.01) | 1.00 (0.99-1.01) |
| **Survival at discharge without severe neonatal morbidity^b^** | | |  |  |
|  | 24-26 w CC (n=170) | 1.16 (0.81-1.67) | 0.89 (0.53-1.49) | 0.77 (0.53-1.13) |
|  | 24-26 w MI (n=181) | 1.12 (0.79-1.60) | 0.89 (0.54-1.49) | 0.77 (0.52-1.13) |
|  | 27-31 w CC (n=294) | **1.09 (1.01-1.19)** | 1.03 (0.88-1.20) | 0.94 (0.87-1.02) |
|  | 27-31 w MI (n=311) | **1.09 (1.01-1.19)** | 1.04 (0.88-1.23) | 0.95 (0.87-1.03) |
| **Survival at 2yo without neurosensory impairment among all fetuses^c^** | | | |  |
|  | 24-26 w CC (n=148) | 1.21 (0.91-1.62) | 1.22 (0.70-2.14) | 0.99 (0.67-1.45) |
|  | 24-26 w MI (n=181) | 1.13 (0.90-1.44) | 1.22 (0.66-2.24) | 0.99 (0.68-1.42) |
|  | 27-31 w CC (n=235) | 1.01 (0.94-1.08) | 0.99 (0.87-1.13) | 0.95 (0.89-1.03) |
|  | 27-31 w MI (n=311) | 1.00 (0.92-1.09) | 1.00 (0.87-1.15) | 0.97 (0.86-1.09) |
| **ASQ below threshold among infants alive at 2yo without neurosensory impairment** | | | |  |
|  | 24-26 w CC (n=72) | 0.85 (0.33-2.17) | 0.51 (0.16-1.64) | 1.68 (0.55-5.12) |
|  | 24-26 w MI (n=119) | 0.93 (0.40-2.17) | 0.62 (0.20-1.87) | 1.18 (0.43-3.19) |
|  | 27-31 w CC (n=170) | 1.04 (0.62-1.74) | 0.60 (0.33-1.10) | 0.90 (0.44-1.84) |
|  | 27-31 w MI (n=301) | 1.01 (0.63-1.60) | 0.77 (0.44-1.35) | 1.17 (0.59-2.30) |

aRR: adjusted risk ratios, CC: complete-cases, MI: multiple imputation, ASQ: Ages and Stages questionnaire, ref: reference, w: weeks of gestation

^a^ No missing data

^b^ Survival at discharge without any of the following: grades III-IV intraventricular hemorrhage, cystic periventricular leukomalacia, stages II-III NEC according to Bell’s staging, stage 3 or greater retinopathy of prematurity or severe bronchopulmonary dysplasia.

^c^ Survival at 2 years of corrected age without cerebral palsy GMFCS levels 2-5 or deafness or blindness

^d^ Any combinations of antibiotics covering >90% of *Escherichia coli* in addition to *Streptococcus agalactiae*

Adjusted risk ratios (aRR) obtained from population-averaged Poisson regression models with robust variance estimation, adjusted for gestational age at PPROM and the type of maternity unit. Associations of antibiotic prophylaxis with intra-uterine infection, early-onset sepsis and late-onset sepsis were not reported as the models did not converge because of small numbers after stratification. There was no case of early-onset sepsis in the associations group, hence this outcome is not reported in the second part of the table. Values in bold are statistically significant.
